# Supplementary figures and images for: Depth effect on the prokaryotic community assemblage associated with sponges from different rocky reefs
Source: PeerJ. 2022 Apr 6;10:e13133. doi: 10.7717/peerj.13133 (PMC8994493; doi:10.7717/peerj.13133)

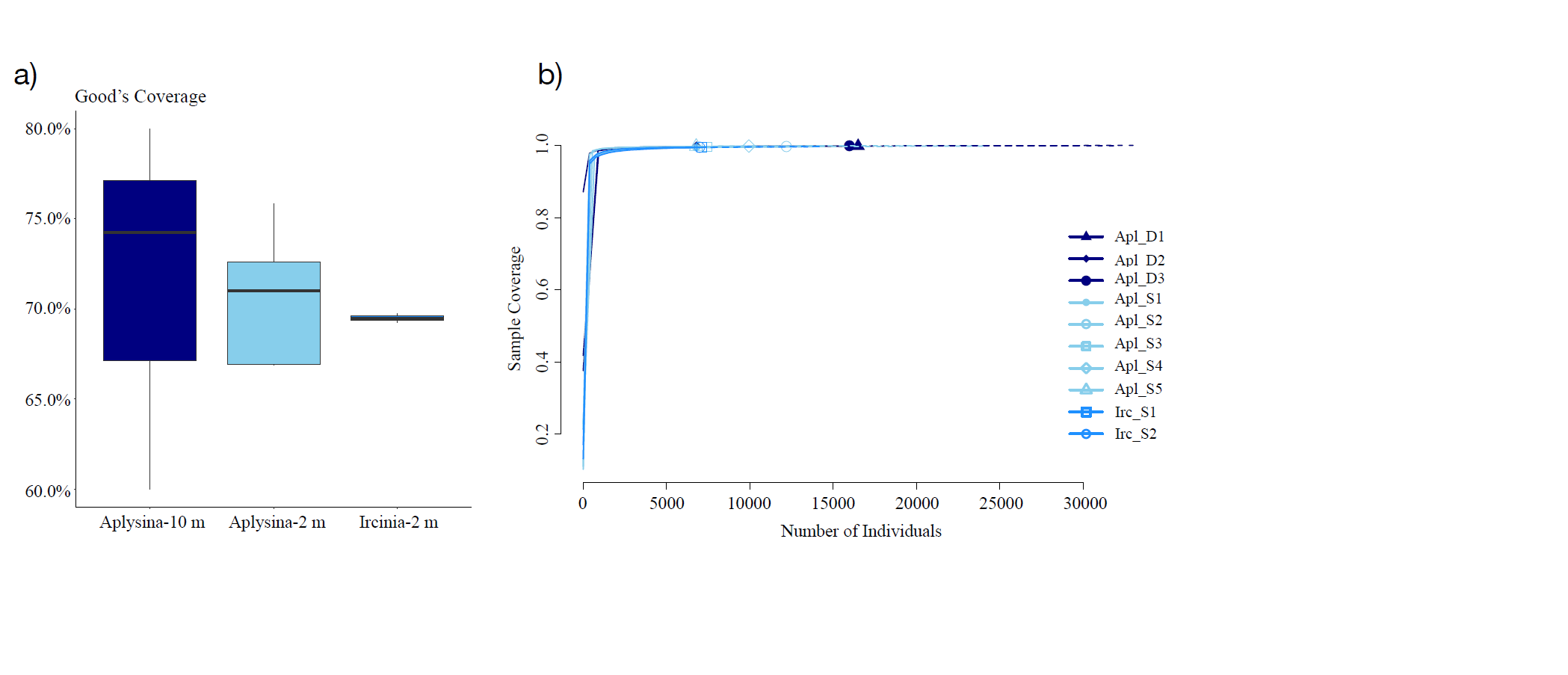

Supplement: Supplemental Information 1 — (a) Good’s coverage, and (b) depth sampling coverage analysis for Aplysina sp and Ircinia sp samples [file peerj-10-13133-s001.png]

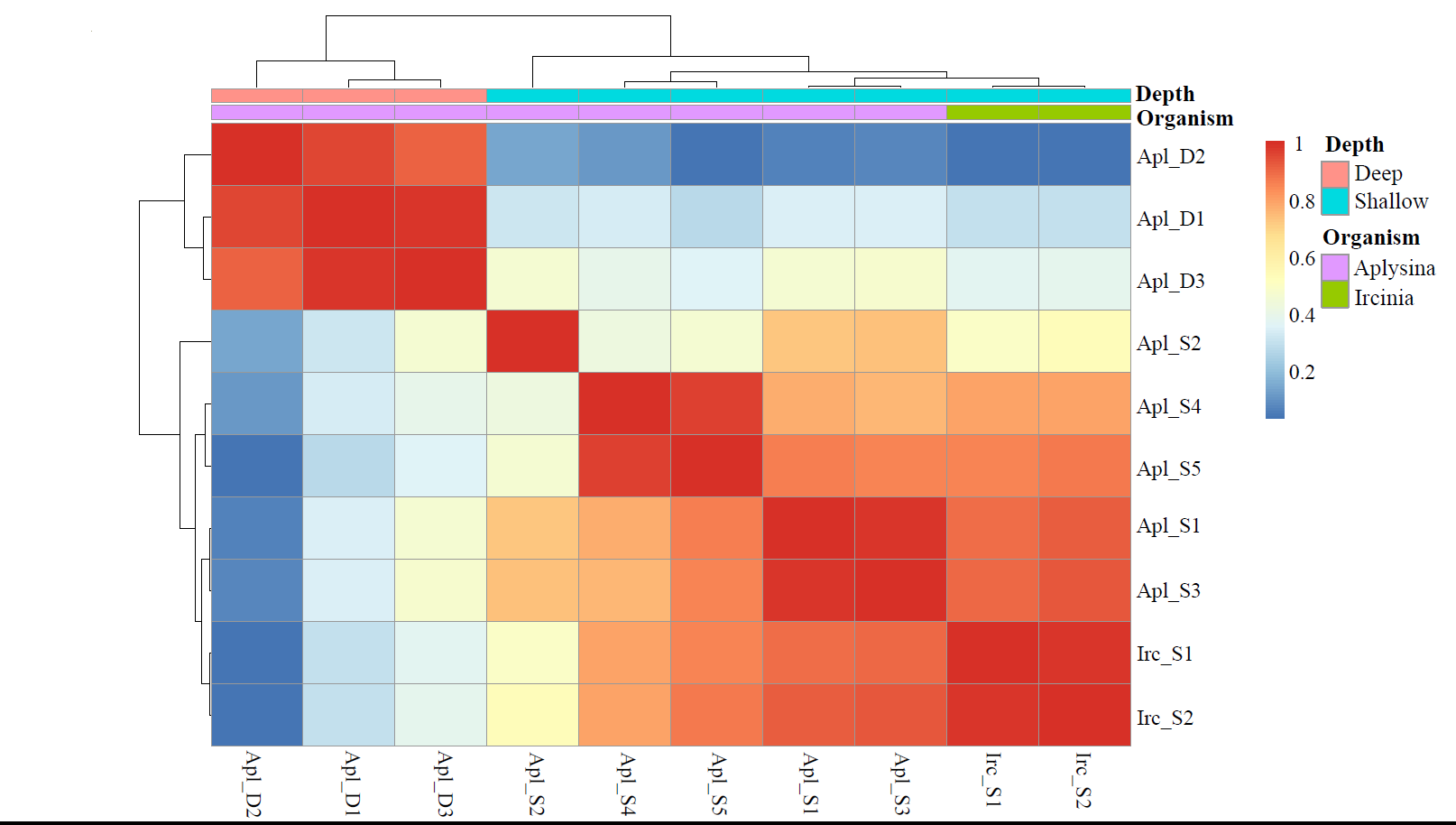

Supplement: Supplemental Information 2 [file peerj-10-13133-s002.png]

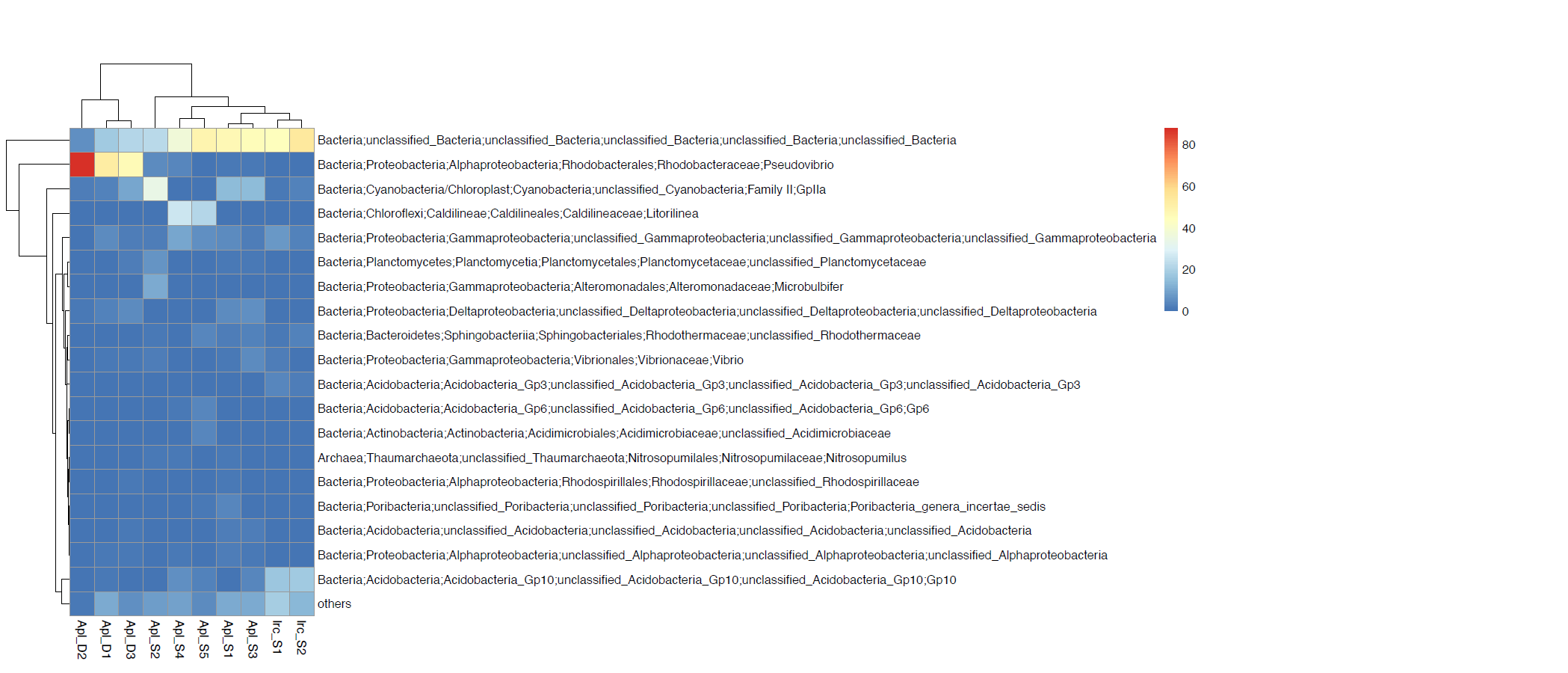

Supplement: Supplemental Information 3 [file peerj-10-13133-s003.png]
